# High visibility first-order subwavelength interference based on light pulse storage via electromagnetically induced transparency

**DOI:** 10.1038/s41598-017-02504-7

**Published:** 2017-05-24

**Authors:** Zhixiang Li, Jianji Liu, Hongming Fan, Jiachen Liu, Guoquan Zhang

**Affiliations:** The MOE Key Laboratory of Weak Light Nonlinear Photonics, School of Physics and TEDA Applied Physics Institute, Nankai University, Tianjin, 300457 China

## Abstract

We achieved high visibility first-order subwavelength interference based on light pulse storage and retrieval technique via electromagnetically induced transparency (EIT) effect in a Pr^3+^:Y_2_SiO_5_ crystal. The interference field distribution of a double-slit was first stored in a Pr^3+^:Y_2_SiO_5_ crystal based on EIT effect, and then it was read out by a spatially modulated readout beam. The retrieved output field is proportional to the product of the input interference field of the double-slit and the spatially modulated readout field. High visibility first-order subwavelength interference with an effective wavelength of *λ*/*n*, where *λ* is the wavelength of the input light field and *n* is any positive integer, can be obtained by designing the spatial modulation structure of the readout field. Experimentally, first-order subwavelength interference with an effective wavelength of *λ*/3 and a visibility of 67% were demonstrated. Such first-order subwavelength interference has important applications on high resolution optical lithography.

## Introduction

Optical interferometric effect is of great importance in both fundamental physics and practical applications such as optical lithography and precision measurement^1^. Due to the wave nature of the light field, the spatial resolution of an optical interferometric system is limited to ~λ/2, the so-called Rayleigh resolution limitation. Therefore, subwavelength interference of light field has attracted much attention due to its ability to break through this spatial resolution limitation, which may result in super-resolved optical lithography and precision measurement.

Various methods were developed to achieve subwavelength interference. Jacobson *et al*.^2^ proposed the concept of photonic de Broglie wave of *n*-bounded photons as a whole with an effective wavelength of *λ*/*n*, and this was experimentally verified in interferometric schemes by using path-entangled two-photon states $({|2,0\rangle }_{A,B}+{|0,2\rangle }_{A,B})/\sqrt{2}$ generated through spontaneous parametric down-conversion process, where the subscription *A* and *B* denote two optical paths in the interferometer^3, 4^. By using the path-entangled noon state $({|n,0\rangle }_{A,B}+{|0,n\rangle }_{A,B})/\sqrt{2}$, subwavelength interference with an effective wavelength of *λ*/*n* and its application to super-resolved phase measurement^5, 6^ and optical lithography^7, 8^ were demonstrated. The interfering fringe visibility of the path-entangled noon state can reach 100% theoretically. The main challenging in the implementation of quantum lithography based on subwavelength interference is the conflicting requirements that the light field should be strong enough to induce efficient multi-photon nonlinearity yet be so weak as to keep the quantum features^9^. Later, subwavelength interference was also reported with classical light sources, but in general the interfering fringe visibility is much lower than that with quantum light sources^10–15^. High visibility subwavelength interference can also be achieved with specially designed classical light sources, for example, by correlating wave vector and frequency with a frequency-selective multi-photon detection that uses Doppleron-type resonances^16^, or using phase-correlated classical light sources in the higher-order optical coherence^17^, however, the difficulty in the availability of multi-photon nonlinear optical materials with efficient high-order optical nonlinearity may become a serious limitation for its practical applications^9^. In addition, multi-photon nonlinear subwavelength interference was demonstrated using coherent lasers by using the phase-shifted multiple exposure technique^18, 19^ and the interference among correlated multiple frequency components^20^ to suppress the low spatial frequency components, but at the expense of reduced fringe visibility. Note that, to achieve a subwavelength spatial resolution with an effective wavelength of *λ*/*n*, *n*-photon nonlinear optical processes are required in general, which, especially in the *n* > 2 cases and with quantum light sources, becomes extremely challenging in consideration of efficient recording materials, and in most case the *n*-photon nonlinear absorption processes are mimicked by *n*-photon coincidence detection technique in the literature^3–14, 16, 17, 19^. Therefore, from the view point of practical applications, high visibility subwavelength interference linear in light intensity, corresponding to the one-photon absorption process, is extremely on demand^9, 21^.

In this paper, we proposed a method to achieve first-order subwavelength interference linear in light intensity based on the light pulse storage and retrieval via electromagnetically induced transparency (EIT) effect in solids. Subwavelength interference of a double-slit with an effective wavelength of *λ*/*n*, where *n* is an arbitrary positive integer, and a theoretical fringe visibility of 100% can be obtained. Experimentally, subwavelength interference with an effective wavelength of *λ*/3 and a fringe visibility of 67% was demonstrated.

## Results

Electromagnetically induced transparency effect is a result of destructive quantum interference among multiple atomic transition paths, which provides a powerful way for coherent manipulation on the interacting light fields and the optical properties of the atomic media^22–27^. In a coherent atomic ensemble driven by a strong coupling beam, the absorption of a probe beam, even resonantly applied on an atomic transition, can be reduced or even eliminated under the EIT condition. According to the Kramers-Kronig relationship, the EIT effect will induce a strong spectral dispersion near the two-photon resonant condition, which was used to slowdown and stop light pulses propagating in the EIT media^28–30^. Storage and retrieval of light pulses carrying optical information^29, 31–33^ or encoded with images^34–36^ were demonstrated based on the EIT effect. Here, we will demonstrate the achievement of high visibility, first-order subwavelength interference through EIT-based light pulse storage and retrieval in solids.

### Light pulse storage and retrieval based on EIT effect

**Figure 1:**
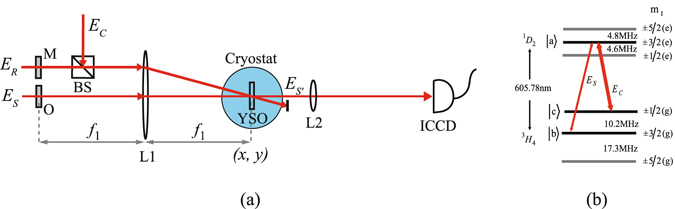
(**a**) The schematic diagram of experimental setup to observe the subwavelength interference of a double-slit through EIT-based light pulse storage and retrieval. Here *E*_*S*_, *E*_*C*_, *E*_*R*_ and *E*_*S*′_ are the input signal, the coupling, the readout and the retrieved output signal beams, respectively. O represents the double-slit, M is the *π*-phase-shifted *n*-slit mask, BS is the beam splitter, L1 and L2 are two lenses, (*x*, *y*) is the transverse coordinates in the rear focal plane of lens L1, ICCD is an intensified charge-coupled device camera. (**b**) The hyperfine energy-level structure of the transition ^1^ *D*_2_ ↔ ^3^ *H*_4_ of Pr^3+^ ion in YSO crystal, where one upper level |*a*〉 and two lower levels |*b*〉 and |*c*〉 form the typical Λ-type three-level configuration employed in the experiment.

For simplicity but without loss of generality, we considered a light pulse storage and retrieval process based on EIT effect in a praseodymium ion doped Y_2_SiO_5_ crystal (Pr:YSO, 0.05%) with a typical Λ-type three-level configuration. The experimental setup is shown in Fig. 1(a). The Pr:YSO crystal was employed as the EIT media, and two lower levels |*b*〉, |*c*〉 and one upper level |*a*〉 of the hyperfine transition ^1^ *D*_2_ ↔ ^3^ *H*_4_ (~605.78 nm) of Pr^3+^ ions, as shown in Fig. 1(b), were chosen to form the Λ-type three-level configuration^37, 38^. The crystal was kept at 3.6 K in a cryostat and was placed in the rear focal plane of lens L1 with a focal length *f*_1_ = 30 cm. Three beams from a Coherent 899 dye ring laser operating at ~605.78 nm served as the signal beam *E*_*S*_, the coupling beam *E*_*C*_ and the readout beam *E*_*R*_ with their respective power of 16 mW, 30 mW and 30 mW. These beams could be temporally modulated in intensity and shifted in frequency independently by the corresponding acousto-optic modulators (not shown here), and they were polarized linearly and in parallel in such a way to guarantee an efficient EIT effect and storage efficiency^39^. A double-slit was placed at the front focal plane of lens L1. A 5-*μ*s signal pulse was transmitted through the double-slit, and the interference field of the double-slit was interacted with the Gaussian coupling beam in the Pr:YSO crystal under the EIT condition, and could be stored in Pr:YSO crystal by adiabatically switching off the coupling field during the interaction. After a certain time delay (~3 *μ*s in our experiments, less than the 30-*μ*s storage time of light pulse in Pr:YSO crystal at 3.6 K), the interference field of the double-slit could be recovered by launching a Gaussian readout beam *E*_*R*_ along the coupling beam under the phase-matching condition. Detailed procedure to prepare the EIT condition and to store and retrieve the signal light pulse can be found in refs 37, 38. The light intensity distribution on the output surface of Pr:YSO crystal was imaged through lens L2 with a focal length *f*_2_ = 10 cm and recorded by an intensified charge-coupled device (ICCD) camera.

### Theoretical analysis

We assume that the light field amplitude in the transverse dimension of the rear focal plane of lens L1, i.e., the transverse distribution of light field in the Pr:YSO crystal, is *E*_*j*_(*x*, *y*) (j = *S*, *C*, *R*, *S*′) for the input signal, the coupling, the readout and the retrieved output signal beams, respectively. Under the paraxial approximation and after a light-pulse storage and retrieval process based on the EIT effect, the field amplitude of the retrieved output signal beam *E*_*S*′_(*x*, *y*) can be written as^38, 39^:

1 $${E}_{S^{\prime} }(x,y)\propto {E}_{S}(x,y){E}_{C}^{\ast }(x,y){E}_{R}(x,y),$$

where the superscript ‘*’ denotes the complex conjugate, and (*x*, *y*) are the transverse coordinates in the rear focal plane of lens L1. Note that the relative light field distribution in the transverse dimension in our case is temporally stable in the rear focal plane of lens L1, therefore we drop the variables *z* and *t* in the expression of light field amplitude for clarity.

For a double-slit with a slit width *a* and a separation distance *d* between two slits and placed on the front focal plane of lens L1, as shown in Fig. 1(a), the interference field of the input signal beam in the Pr:YSO crystal can be expressed as

2 $${E}_{S}(x)\propto \,\cos (\frac{\pi dx}{\lambda {f}_{1}}){\rm{sinc}}(\frac{\pi ax}{\lambda {f}_{1}}),$$

where the term cos(*πdx*/*λf*_1_) is a result of interference of light from two slits with its interference period of Λ_*int*_ = *λf*_1_/*d*, while the term sinc(*πax*/*λf*_1_) describes the diffraction effect of a single slit. This interference pattern is then stored in Pr:YSO crystal based on the EIT effect by adiabatically switching off the Gaussian coupling beam, which can be approximately characterized by a constant field amplitude *E*_*C*_ in Eq. (1) when its spot size is much larger than those of the input signal and readout beams. According to Eq. (1), the interference field expressed by Eq. (2) can then be recovered by launching a Gaussian readout beam propagating along the coupling beam.

Interestingly, one notes that, by reading out the stored interference light field expressed by Eq. (2) with a spatially modulated readout field *E*_*R*_ in a formula proportional to sin(*πdx*/*λf*_1_), it is possible to double the spatial frequency of the interference pattern according to Eq. (1). The simplest way to obtain such a spatially modulated readout beam is to interfere two coherent beams. Here, for the purpose to achieve subwavelength interference with subtle spatial structure, we propose a novel way to produce the spatially modulated readout beam with the required transverse spatial distribution of light field. We let the readout beam *E*_*R*_ transmit through another double-slit mask M with the same slit width *a* and separation distance *d* as the one used to modulate the input signal beam, and the double-slit mask M is placed also on the front focal plane of lens L1 but deviated transversely from the optical axis of the experimental setup, as shown in Fig. 1(a). By introducing a *π*-phase-shift on one slit of the double-slit M, one can produce a diffraction light field ${E}_{R}(x)\propto \,\sin (\pi dx/\lambda {f}_{1}){\rm{sinc}}(\pi ax/\lambda {f}_{1})$ in the rear focal plane of lens L1, which is phase-shifted by *π*/2 with respect to *E*_*S*_(*x*). Note that there is an additional diffraction term sinc(*πax*/*λf*_1_) associated with *E*_*R*_(*x*), which will modify the envelope of the retrieved output signal interference pattern, as we will shown in the following.

According to Eq. (1), the retrieved output signal field can then be expressed as

3 $${E}_{S^{\prime} }(x)\propto \sin (\frac{\pi dx}{(\lambda /2){f}_{1}}){{\rm{s}}{\rm{i}}{\rm{n}}{\rm{c}}}^{2}(\frac{\pi ax}{\lambda {f}_{1}}).$$

One sees that, as compared to the interference pattern of the input signal field *E*_*S*_(*x*) in Eq. (2), the spatial frequency of the interference pattern of *E*_*S*′_(*x*) in Eq. (3) is doubled, and the interference period Λ_*int*_ = *λf*_*1*_ */(2d)* is now only half of that of the input signal field *E*_*S*_, indicating an effective wavelength of *λ*/2. The intensity interference pattern of the retrieved output signal beam is now enveloped by sinc^4^(*πax*/*λf*_1_), which is different from that of the input signal interference pattern enveloped by sinc^2^(*πax*/*λf*_1_).

The spatial frequency of the retrieved output signal field can be further increased with an effective wavelength of *λ*/*n*, where *n* is an integer. In general, one can use a *n*-slit mask M with the same slit width *a* and separation distance *d* as those of the double-slit O used to produce the input signal interference pattern, and sets the light fields transmitting through two neighboring slits of *n*-slit mask M out of phase, i.e., introduces a *π*-phase-shift between two nearest slits of *n*-slit mask M. We call such kind of mask as *π*-phase-shifted *n*-slit mask. Through similar light pulse storage and retrieval procedure to double the spatial frequency of the interference pattern as described above, but replacing the *π*-phase-shifted double-slit mask with the *π*-phase-shifted *n*-slit mask in the optical path of readout beam *E*_*R*_, one can obtain a retrieved output signal field as

4 $${E}_{S^{\prime} }(x)\propto \,\sin (\frac{\pi dx}{(\lambda /n){f}_{1}}){{\rm{sinc}}}^{2}(\frac{\pi ax}{\lambda {f}_{1}})$$

when *n* is an even integer, or

5 $${E}_{S^{\prime} }(x)\propto \,\cos (\frac{\pi dx}{(\lambda /n){f}_{1}}){{\rm{sinc}}}^{2}(\frac{\pi ax}{\lambda {f}_{1}})$$

when *n* is an odd integer. It is evident that the interference period of the retrieved output signal field is only one *n*th of that of the input signal field, producing a subwavelength interference with an effective wavelength of *λ*/*n*.

### Experimental verification

**Figure 2:**
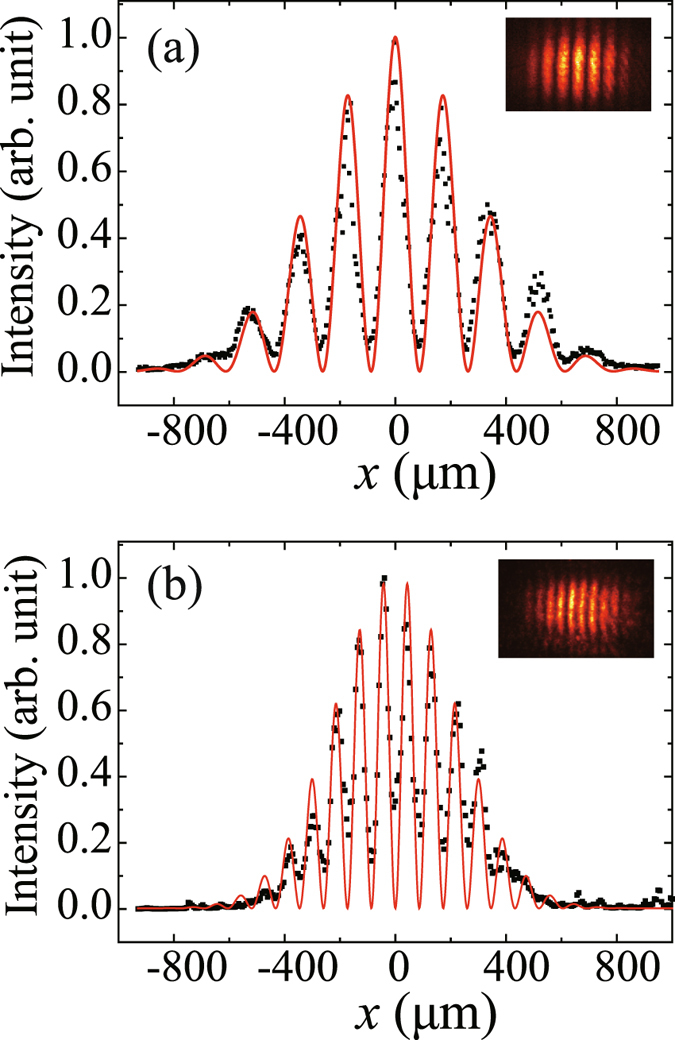
The transverse intensity profiles of the interference pattern for the input signal field *E*_*S*_ (**a**) and the retrieved output signal field *E*_*S*′_ with an effective wavelength of *λ*/2 (**b**) in the rear focal plane of lens L1. The slit width *a* and the separation distance *d* of the double-slit are 200 *μ*m and 1000 *μ*m, respectively. The insets show the corresponding intensity distribution recorded by ICCD through lens L2. The black squares are the experimental data, and the red solid curves are theoretical simulation using Eq. (2) for (**a**) and Eq. (3) for (**b**), respectively, with experimental parameters and without any fitting parameter.

Figure 2 shows the transverse intensity profiles of the interference patterns in the rear focal plane of lens L1 for the case of a double-slit with *a* = 200 *μ*m and *d* = 1000 *μ*m, where Fig. 2(a) is the interference pattern of the input signal field *E*_*S*_, while Fig. 2(b) is the one for the retrieved output signal field *E*_*S*′_ with a readout beam transmitting through a *π*-phase-shifted double-slit M of the same slit width *a* = 200 *μ*m and separation distance *d* = 1000 *μ*m. The interference period in Fig. 2(a) was measured to be 182 *μ*m and that in Fig. 2(b) was measured to be 91 *μ*m with a visibility of 78%, achieving a spatial frequency doubling for the retrieved output signal field *E*_*S*′_ as compared to the input signal field *E*_*S*_. Note that the interference period of *E*_*S*′_ with a Gaussian readout beam was the same as that in Fig. 2(a). This verified experimentally the first-order subwavelength interference with an effective wavelength of *λ*/2 through the EIT-based light pulse storage and retrieval process according to Eq. (3). The red solid curves in Figs. 2(a) and 2(b) were simulated according to Eqs. (2) and (3), respectively, with the experimental parameters *a* = 200 *μ*m, *d* = 1000 *μ*m, *f*_1_ = 30 cm and *λ* = 605.78 nm, and no adjustable fitting parameter was employed. Good agreement is achieved between the theoretical prediction and the experimental measurement.

**Figure 3:**
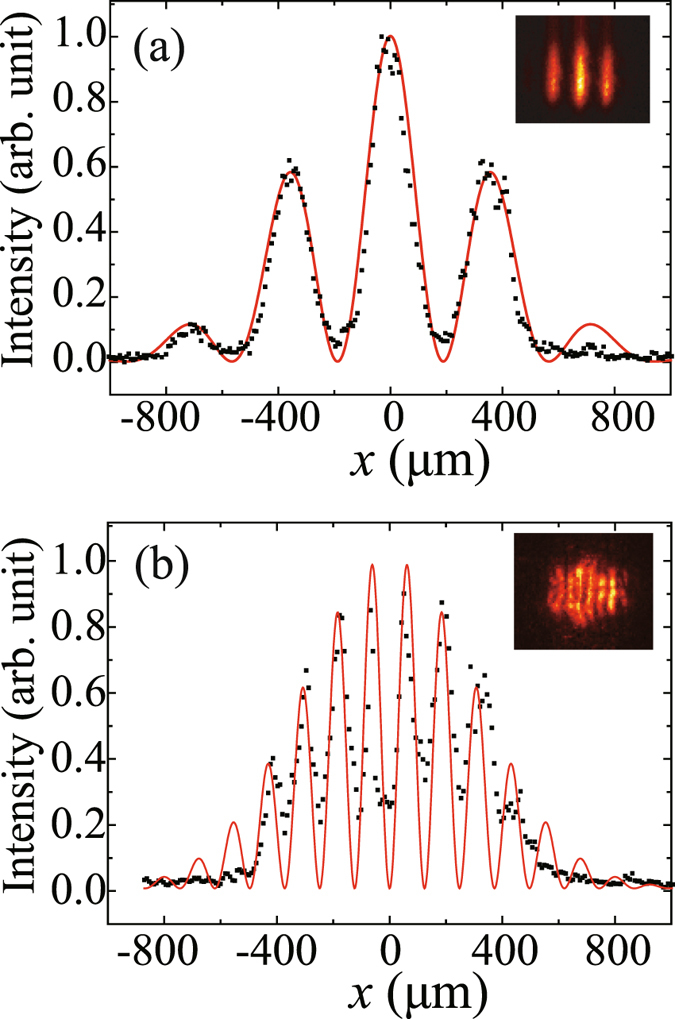
The transverse intensity profiles of the interference pattern for the input signal field *E*_*S*_ (**a**) and the retrieved output signal field *E*_*S*′_ with an effective wavelength of *λ*/3 (**b**) in the rear focal plane of lens L1. The slit width *a* and the separation distance *d* of the double-slit are 200 *μ*m and 500 *μ*m, respectively. The insets show the corresponding intensity distribution recorded by ICCD through lens L2. The black squares are the experimental data, and the red solid curves are theoretical simulation using Eq. (2) for (**a**) and Eq. (5) for (**b**), respectively, with experimental parameters and without any fitting parameter.

Figure 3 shows the subwavelength interference with an effective wavelength of *λ*/3, where a double-slit with *d* = 500 *μ*m and *a* = 200 *μ*m was employed, and a *π*-phase-shifted three-slit mask with the same *d* = 500 *μ*m and *a* = 200 *μ*m was inserted in the optical path of readout beam *E*_*R*_. Similarly, Figs. 3(a) and 3(b) are the interference patterns in the rear focal plane of lens L1 for the input signal field and the retrieved output signal field with the *π*-phase-shifted three-slit mask in the optical path of the readout beam, respectively. As expected, the spatial frequency of the interference pattern in Fig. 3(b) is tripled as compared to that in Fig. 3(a), indicating a subwavelength interference with an effective wavelength of *λ*/3. The visibility of the first-order subwavelength interference was measured to be 67%, which is lower than the theoretically predicted 100%. This is mainly due to the field profile mismatching between the signal field *E*_*S*_(*x*, *y*) and the readout field *E*_*R*_(*x*, *y*), which could originate from structure parameter deviation of the fabricated *π*-phase-shifted three-slit mask M (see section Methods for the fabrication process of mask M), especially the phase-shift between the neighboring slits, as compared to the ideal one. The vibration of the cryostat, which was measured to be up to ~3 *μ*m in the experiment, is another important contribution to the observed reduction in the fringe visibility. Note that, although the experimentally measured fringe visibility is lower than the theoretically predicted one in our case, it is still much higher than those achieved in subwavelength interference with classical light based on photon correlation measurement^10–15, 18–20^.

It is evident that, according to Eqs. (4) and (5), subwavelength interference with an effective wavelength of *λ*/*n* and a theoretical visibility of 100% can be achieved through similar EIT-based light pulse storage and retrieval process with the assistance of the *π*-phase-shifted *n*-slit mask during the readout of the stored interfering light field of the double-slit. Therefore, interference fringe patterns with period smaller than *λ*/2, which breaks through the diffraction limit of light, is theoretically possible. This seems to be surprising, but actually it is understandable because the observed interference fringe is a result of nonlinear mixing process between the signal field and the readout field through the EIT-based atomic coherence gratings. The key point here is that such a nonlinear mixing process results in a spatial frequency doubling or tripling effect, which leads to the observed subwavelength interference. It can be easily confirmed that one cannot get such subwavelength interference pattern by simply superposing the signal field *E*_*S*_(*x*, *y*) and the readout field *E*_*R*_(*x*, *y*) in linear space. We notice that the period of the fringe pattern in Figs. 2(b) and 3(b) is still much larger than the diffraction limit of light. In our current experimental setup, because of the bulky volume of cryostat and the spatial resolution limitation of ICCD (with a pixel size of 13 *μ*m × 13 *μ*m), the spatial resolution of the optical detection system (consisting of lens L2 and ICCD) was limited to ~3 *μ*m. New detection system with high spatial resolution is needed to observe subwavelength interference fringes with a period less than *λ*/2, which is in progress in our laboratory.

## Discussion

Subwavelength interference in high-order optical coherence with an effective wavelength of *λ*/*n* was reported for both quantum light sources and classical light sources. In general, *n*-photon coincidence detection, corresponding to *n*-photon nonlinear optical process, is performed experimentally instead of a real *n*-photon nonlinear recording process because of the challenging requirement on the optical nonlinear sensitivity of the recording materials, especially when quantum light sources such as noon state $({|\mathrm{n,}0\rangle }_{A,B}+{|\mathrm{0,}n\rangle }_{A,B})/\sqrt{2}$ are used^3–9^. Classical light sources such as thermal light were also used to generate second-order subwavelength interference, but usually the two coincidence detectors are scanning in opposite directions which is not suitable for practical applications such as optical lithography^10, 11^. Subwavelength interference can also be achieved with phased correlated light sources^13–15, 17^ or independent light sources with detectors positioned at specific magic angles^12^, but usually the fringe visibility is significantly reduced as compared to those of quantum light sources. In certain cases, the fringe visibility can be improved by using high-order optical coherence^16, 17^, which, however, requires sensitive *n*-photon high-order optical nonlinearity in practical applications. In our case, the subwavelength interference is achieved by generating the high-order harmonics by, for example, doubling or tripling the spatial frequency of the input interference fringes via an EIT-based light pulse storage and retrieval process, and it is a kind of subwavelength interference in the first-order optical coherence, i.e., the light intensity interference fringes, which corresponds to the one-photon nonlinear optical process and therefore is compatible to the current optical lithography platform^21^. Moreover, the fringe visibility can reach 100% theoretically no matter how fine the fringe structure is.

We note that resonant interferometric subwavelength localization of atomic excitation was suggested based on coherent population trapping in an atomic ensemble with *N* × Λ energy-level structure, where the key is to cancel the lower spatial harmonics with a suitable choice of the relative phase shifts among *N* pairs of coupling and signal standing waves^40^. One sees here that, to achieve an interferometric subwavelength localization of atomic excitation with a wave number 2*π*/(*λ*/2*N*), atomic ensembles with special *N* × Λ energy-level structure are required in this technique. Subwavelength localization of atomic excitation was reported under the EIT condition, where the underlying mechanism is the sensitivity of dark state of EIT to the coupling beam intensity^41–45^. This results in a nonlinear dependence of the population of atomic level on the spatial intensity distribution of the coupling beam, especially at the intensity nodes of the coupling beam where the excitation is tightly localized, and it is observable with a post-selection measurement, for example, through optical fluorescence. Also, due to the sensitive dependence of the refractive index and absorption of the EIT media on the coupling beam intensity, optical image transfer from the coupling beam to the probe beam beyond the diffraction limit was reported under the EIT condition^46, 47^. One sees that all these above-mentioned techniques are based on the subwavelength localization of atomic excitation. Although the subwavelength interference fringes reported in this paper is also based on the EIT effect, the involved mechanism is totally different from those mentioned above. In our case, there is no subwavelength localization of atomic excitation during the interaction between the coupling and the signal fields, the subwavelength interference fringes are the product of the normal interfering signal field and the matched spatially modulated readout field, which is achieved through an EIT-based light pulse storage and retrieval process. Note that in our case the coupling field is a spatially homogeneous field and the input interference fringe of the signal field is normal and obeys the diffraction limit of light wave, it is the readout field that is spatially modulated to match the generation of desired high-order spatial frequency components of the input interference fringes of the signal field. Furthermore, no post-selection measurement is necessary.

## Methods

### Preparation of the *π*-phase-shifted *n*-slit mask

The *π*-phase-shifted *n*-slit mask is actually composed of two parts. One part is the transmitting *n*-slit metallic mask with a slit width *a* and a separation distance *d* between two neighboring slits. The other part is a transmitting spatial light modulator (SLM) operating in phase-only mode. The *n*-slit metallic mask is placed just before the SLM, and the phase of the light field transmitting through each slit of the metallic mask is modulated by the SLM.
